# *N*-(3-Methyl­benzo­yl)-2-nitro­benzene­sulfonamide

**DOI:** 10.1107/S160053681200164X

**Published:** 2012-01-18

**Authors:** P. A. Suchetan, Sabine Foro, B. Thimme Gowda

**Affiliations:** a Department of Chemistry, Mangalore University, Mangalagangotri 574 199, Mangalore, India; b Institute of Materials Science, Darmstadt University of Technology, Petersenstrasse 23, D-64287 Darmstadt, Germany

## Abstract

In the title compound, C_14_H_12_N_2_O_5_S, the conformation between the N—H group and the *ortho*-nitro group in the sulfonyl benzene ring is *syn* and that between the C=O and *meta*-methyl groups in the benzoyl ring is *anti*. The mol­ecule is twisted at the S—N bond with a torsion angle of 64.3 (2)°. The dihedral angle between the sulfonyl benzene ring and the —SO_2_—NH—C—O segment is 75.73 (7)° and that between the sulfonyl and benzoyl benzene rings is 89.5 (1)°. The crystal structure features inversion-related dimers linked by pairs of N—H⋯O(S) hydrogen bonds.

## Related literature

For studies, including by our group, on the effects of substituents on the structures and other aspects of *N*-(ar­yl)-amides, see: Bowes *et al.* (2003 ▶); Gowda *et al.* (1999 ▶, 2003 ▶), on *N*-(ar­yl)-methane­sulfonamides, see: Gowda *et al.* (2007 ▶), on *N*-(ar­yl)-aryl­sulfonamides, see: Shetty & Gowda (2005 ▶), on *N*-(substitutedbenzo­yl)-aryl­sulfonamides, see: Suchetan *et al.* (2012 ▶), on *N*-chloro­aryl­amides, see: Jyothi & Gowda (2004 ▶) and on *N*-bromo­aryl­sulfonamides, see: Usha & Gowda (2006 ▶).
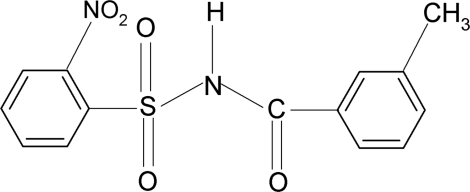


## Experimental

### 

#### Crystal data

C_14_H_12_N_2_O_5_S
*M* *_r_* = 320.32
Orthorhombic, 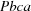

*a* = 12.227 (1) Å
*b* = 12.854 (1) Å
*c* = 18.317 (2) Å
*V* = 2878.8 (5) Å^3^
*Z* = 8
Mo *K*α radiation
μ = 0.25 mm^−1^
*T* = 293 K
0.48 × 0.44 × 0.32 mm

#### Data collection

Oxford Diffraction Xcalibur diffractometer with a Sapphire CCD detector
Absorption correction: multi-scan (*CrysAlis RED*; Oxford Diffraction, 2009 ▶) *T*_min_ = 0.889, *T*_max_ = 0.924
7423 measured reflections
2936 independent reflections
2306 reflections with *I* > 2σ(*I*)
*R*_int_ = 0.019

#### Refinement

*R*[*F* ^2^ > 2σ(*F* ^2^)] = 0.044
*wR*(*F* ^2^) = 0.124
*S* = 1.04
2936 reflections
203 parameters
1 restraint
H atoms treated by a mixture of independent and constrained refinement
Δρ_max_ = 0.38 e Å^−3^
Δρ_min_ = −0.33 e Å^−3^

### 

Data collection: *CrysAlis CCD* (Oxford Diffraction, 2009 ▶); cell refinement: *CrysAlis RED* (Oxford Diffraction, 2009 ▶); data reduction: *CrysAlis RED*; program(s) used to solve structure: *SHELXS97* (Sheldrick, 2008 ▶); program(s) used to refine structure: *SHELXL97* (Sheldrick, 2008 ▶); molecular graphics: *PLATON* (Spek, 2009 ▶); software used to prepare material for publication: *SHELXL97*.

## Supplementary Material

Crystal structure: contains datablock(s) I, global. DOI: 10.1107/S160053681200164X/bq2333sup1.cif

Structure factors: contains datablock(s) I. DOI: 10.1107/S160053681200164X/bq2333Isup2.hkl

Supplementary material file. DOI: 10.1107/S160053681200164X/bq2333Isup3.cml

Additional supplementary materials:  crystallographic information; 3D view; checkCIF report

## Figures and Tables

**Table 1:** Hydrogen-bond geometry (Å, °)

*D*—H⋯*A*	*D*—H	H⋯*A*	*D*⋯*A*	*D*—H⋯*A*
N1—H1*N*⋯O2^i^	0.85 (2)	2.43 (2)	3.232 (3)	157 (2)

Symmetry code: (i) 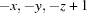.

**Fig. 1.**
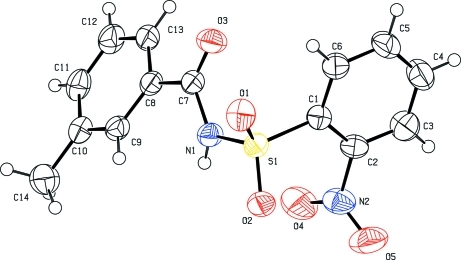
Molecular structure of the title compound, showing the atom-labeling scheme. Displacement ellipsoids are drawn at the 50% probability level.

**Fig. 2.**
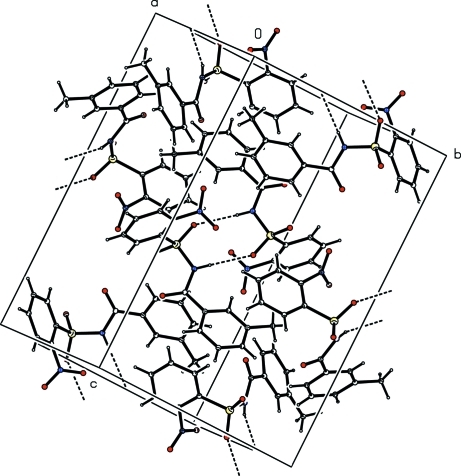
Molecular packing in the title compound. Hydrogen bonds are shown as dashed lines.

## supplementary crystallographic information

### Comment

Diaryl acylsulfonamides are known as potent antitumor agents. As part of our studies on the substituent effects on the structures and other aspects of *N*-(aryl)-amides (Bowes *et al.*, 2003; Gowda *et al.*, 1999, 2003), *N*-(aryl)-methanesulfonamides (Gowda *et al.*, 2007), *N*-(aryl)-arylsulfonamides (Shetty & Gowda, 2005); *N*-(substitutedbenzoyl)-arylsulfonamides (Suchetan *et al.*, 2012); *N*-chloroarylsulfonamides (Jyothi & Gowda, 2004) and *N*-bromoarylsulfonamides (Usha & Gowda, 2006), in the present work, the crystal structure of *N*-(3-methylbenzoyl)-2-nitrobenzenesulfonamide (I) has been determined (Fig.1).

The conformation between the N—H and C=O bonds in the C—SO_2_—NH—C(O) segment is *anti* and the N—C bond in the segment has *gauche* torsion with respect to the S═O bonds (Fig.1), similar to that observed in *N*-(2-chlorobenzoyl)- 2-nitrobenzenesulfonamide (II)(Suchetan *et al.*, 2012). In (I), the conformation between the N—H bond and the *ortho*-nitro group in the sulfonyl benzene ring is *syn*, similar to that observed in (II). Further, the conformation of the C═O is *anti* to the *meta*-methyl group in the benzoyl ring, similar to that observed between the C═O and the *ortho*-Cl atom in (II).

The molecule is twisted at the S—N bond with the torsional angle of 64.32 (20)°, compared to the value of -59.68 (17)° in (II).

The dihedral angle between the sulfonyl benzene ring and the —SO_2_—NH—C—O segment is 75.7 (1)°, compared to the value of 77.5 (1)° in (II). Furthermore, the dihedral angle between the sulfonyl and the benzoyl benzene rings is 89.5 (1)°, compared to the value of 71.2 (1)° in (II).

In the crystal, the intermolecular N–H···O (S) hydrogen bonds (Table 1) link the molecules into dimeric chains. Part of the crystal structure is shown in Fig. 2.

### Experimental

The title compound was prepared by refluxing a mixture of *m*-methylbenzoic acid (0.02 mole), 2-nitrobenzenesulfonamide (0.02 mole) and excess phosphorous oxychloride for 3 h on a water bath. The resultant mixture was cooled and poured into crushed ice. The solid, *N*-(3-methylbenzoyl)-2-nitrobenzenesulfonamide, obtained was filtered, washed thoroughly with water and then dissolved in sodium bicarbonate solution. The compound was later reprecipitated by acidifying the filtered solution with dilute HCl. It was filtered, dried and recrystallized.

Prism like colourless single crystals of the title compound used in X-ray diffraction studies were obtained by slow evaporation of its toluene solution at room temperature.

### Refinement

The H atom of the NH group was located in a difference map and later restrained to N—H = 0.86 (2) %A. The other H atoms were positioned with idealized geometry using a riding model with the aromatic C—H = 0.93 Å and the methyl C—H = 0.93 Å. All H atoms were refined with isotropic displacement parameters set at 1.2 *U*_eq_(C-aromatic, N) and 1.5 *U*_eq_(*C*-methyl).

### Crystal data

**Table:**

C_14_H_12_N_2_O_5_S	*F*(000) = 1328
*M**_r_* = 320.32	*D*_x_ = 1.478 Mg m^−^^3^
Orthorhombic, *P**b**c**a*	Mo *K*α radiation, λ = 0.71073 Å
Hall symbol: -P 2ac 2ab	Cell parameters from 2012 reflections
*a* = 12.227 (1) Å	θ = 2.6–27.8°
*b* = 12.854 (1) Å	µ = 0.25 mm^−^^1^
*c* = 18.317 (2) Å	*T* = 293 K
*V* = 2878.8 (5) Å^3^	Prism, colorless
*Z* = 8	0.48 × 0.44 × 0.32 mm

### Data collection

**Table:**

Oxford Diffraction Xcalibur diffractometer with a Sapphire CCD detector	2936 independent reflections
Radiation source: fine-focus sealed tube	2306 reflections with *I* > 2σ(*I*)
graphite	*R*_int_ = 0.019
Rotation method data acquisition using ω scans	θ_max_ = 26.4°, θ_min_ = 2.6°
Absorption correction: multi-scan (*CrysAlis RED*; Oxford Diffraction, 2009)	*h* = −15→8
*T*_min_ = 0.889, *T*_max_ = 0.924	*k* = −8→16
7423 measured reflections	*l* = −21→22

### Refinement

**Table:**

Refinement on *F*^2^	Primary atom site location: structure-invariant direct methods
Least-squares matrix: full	Secondary atom site location: difference Fourier map
*R*[*F*^2^ > 2σ(*F*^2^)] = 0.044	Hydrogen site location: inferred from neighbouring sites
*wR*(*F*^2^) = 0.124	H atoms treated by a mixture of independent and constrained refinement
*S* = 1.04	*w* = 1/[σ^2^(*F*_o_^2^) + (0.0579*P*)^2^ + 1.5722*P*] where *P* = (*F*_o_^2^ + 2*F*_c_^2^)/3
2936 reflections	(Δ/σ)_max_ = 0.001
203 parameters	Δρ_max_ = 0.38 e Å^−^^3^
1 restraint	Δρ_min_ = −0.33 e Å^−^^3^

### Special details

**Table:**

Experimental. CrysAlis RED (Oxford Diffraction, 2009) Empirical absorption correction using spherical harmonics, implemented in SCALE3 ABSPACK scaling algorithm.
Geometry. All e.s.d.'s (except the e.s.d. in the dihedral angle between two l.s. planes) are estimated using the full covariance matrix. The cell e.s.d.'s are taken into account individually in the estimation of e.s.d.'s in distances, angles and torsion angles; correlations between e.s.d.'s in cell parameters are only used when they are defined by crystal symmetry. An approximate (isotropic) treatment of cell e.s.d.'s is used for estimating e.s.d.'s involving l.s. planes.
Refinement. Refinement of *F*^2^ against ALL reflections. The weighted *R*-factor *wR* and goodness of fit *S* are based on *F*^2^, conventional *R*-factors *R* are based on *F*, with *F* set to zero for negative *F*^2^. The threshold expression of *F*^2^ > σ(*F*^2^) is used only for calculating *R*-factors(gt) *etc*. and is not relevant to the choice of reflections for refinement. *R*-factors based on *F*^2^ are statistically about twice as large as those based on *F*, and *R*- factors based on ALL data will be even larger.

### Fractional atomic coordinates and isotropic or equivalent isotropic displacement parameters (Å^2^)

**Table:**

	*x*	*y*	*z*	*U*_iso_*/*U*_eq_	
C1	−0.01166 (16)	0.27278 (16)	0.42995 (11)	0.0350 (5)	
C2	−0.08805 (18)	0.29514 (18)	0.48387 (12)	0.0388 (5)	
C3	−0.1383 (2)	0.3902 (2)	0.48790 (14)	0.0527 (6)	
H3	−0.1875	0.4044	0.5253	0.063*	
C4	−0.1152 (2)	0.4641 (2)	0.43633 (15)	0.0570 (7)	
H4	−0.1501	0.5283	0.4381	0.068*	
C5	−0.0406 (2)	0.4437 (2)	0.38192 (15)	0.0555 (7)	
H5	−0.0253	0.4942	0.3470	0.067*	
C6	0.0114 (2)	0.34880 (18)	0.37891 (13)	0.0458 (6)	
H6	0.0624	0.3358	0.3423	0.055*	
C7	−0.04990 (18)	0.07627 (17)	0.31441 (11)	0.0374 (5)	
C8	−0.12208 (17)	−0.00987 (17)	0.29044 (11)	0.0360 (5)	
C9	−0.12101 (18)	−0.10611 (17)	0.32424 (11)	0.0382 (5)	
H9	−0.0711	−0.1183	0.3616	0.046*	
C10	−0.19217 (19)	−0.18475 (18)	0.30397 (12)	0.0410 (5)	
C11	−0.2658 (2)	−0.16427 (19)	0.24824 (13)	0.0478 (6)	
H11	−0.3160	−0.2150	0.2345	0.057*	
C12	−0.2657 (2)	−0.0692 (2)	0.21280 (13)	0.0508 (6)	
H12	−0.3144	−0.0575	0.1747	0.061*	
C13	−0.19427 (19)	0.00806 (19)	0.23328 (12)	0.0439 (5)	
H13	−0.1943	0.0717	0.2091	0.053*	
C14	−0.1881 (2)	−0.2872 (2)	0.34183 (15)	0.0585 (7)	
H14A	−0.1384	−0.2832	0.3823	0.070*	
H14B	−0.1636	−0.3397	0.3083	0.070*	
H14C	−0.2598	−0.3048	0.3593	0.070*	
N1	−0.01587 (16)	0.06904 (15)	0.38733 (10)	0.0396 (4)	
H1N	−0.0492 (18)	0.0295 (18)	0.4169 (12)	0.048*	
N2	−0.11943 (17)	0.21727 (18)	0.53943 (12)	0.0520 (5)	
O1	0.15719 (13)	0.17155 (13)	0.38424 (9)	0.0509 (4)	
O2	0.07425 (14)	0.11701 (13)	0.50054 (9)	0.0467 (4)	
O3	−0.02247 (15)	0.14867 (13)	0.27686 (9)	0.0522 (4)	
O4	−0.1626 (2)	0.13838 (17)	0.51818 (12)	0.0779 (6)	
O5	−0.10064 (18)	0.23912 (19)	0.60303 (10)	0.0743 (6)	
S1	0.06292 (4)	0.15463 (4)	0.42754 (3)	0.03641 (18)	

### Atomic displacement parameters (Å^2^)

**Table:**

	*U*^11^	*U*^22^	*U*^33^	*U*^12^	*U*^13^	*U*^23^
C1	0.0315 (10)	0.0356 (11)	0.0379 (10)	−0.0050 (9)	−0.0016 (8)	0.0003 (9)
C2	0.0348 (11)	0.0432 (12)	0.0383 (11)	−0.0042 (10)	0.0017 (9)	0.0074 (10)
C3	0.0457 (14)	0.0565 (15)	0.0558 (14)	0.0051 (12)	0.0116 (11)	0.0021 (12)
C4	0.0618 (17)	0.0420 (14)	0.0672 (16)	0.0094 (13)	0.0066 (13)	0.0048 (12)
C5	0.0707 (17)	0.0399 (13)	0.0559 (14)	−0.0009 (13)	0.0121 (13)	0.0125 (12)
C6	0.0521 (14)	0.0427 (13)	0.0427 (12)	−0.0032 (11)	0.0105 (10)	0.0024 (10)
C7	0.0424 (12)	0.0369 (11)	0.0328 (10)	0.0049 (10)	−0.0020 (9)	−0.0024 (9)
C8	0.0386 (11)	0.0380 (11)	0.0315 (10)	0.0037 (10)	−0.0018 (8)	−0.0051 (9)
C9	0.0399 (12)	0.0415 (12)	0.0331 (10)	0.0026 (10)	−0.0059 (9)	−0.0043 (9)
C10	0.0441 (12)	0.0414 (12)	0.0374 (11)	−0.0008 (10)	0.0012 (9)	−0.0078 (10)
C11	0.0454 (13)	0.0486 (13)	0.0492 (13)	−0.0030 (11)	−0.0073 (10)	−0.0171 (11)
C12	0.0526 (15)	0.0542 (15)	0.0457 (12)	0.0088 (12)	−0.0184 (11)	−0.0125 (12)
C13	0.0545 (14)	0.0402 (12)	0.0371 (11)	0.0100 (11)	−0.0077 (10)	−0.0051 (10)
C14	0.0701 (17)	0.0478 (14)	0.0577 (15)	−0.0141 (13)	−0.0055 (13)	−0.0004 (13)
N1	0.0475 (11)	0.0375 (10)	0.0337 (9)	−0.0093 (9)	−0.0039 (8)	0.0002 (8)
N2	0.0445 (12)	0.0611 (14)	0.0505 (12)	0.0044 (11)	0.0119 (9)	0.0157 (11)
O1	0.0370 (9)	0.0558 (10)	0.0600 (10)	−0.0012 (8)	0.0064 (8)	−0.0048 (8)
O2	0.0538 (10)	0.0472 (9)	0.0392 (8)	0.0012 (8)	−0.0141 (7)	0.0009 (7)
O3	0.0684 (11)	0.0454 (9)	0.0428 (9)	−0.0089 (9)	−0.0063 (8)	0.0099 (8)
O4	0.0936 (16)	0.0595 (13)	0.0806 (14)	−0.0224 (12)	0.0100 (12)	0.0177 (11)
O5	0.0813 (14)	0.0986 (17)	0.0429 (10)	0.0126 (13)	0.0139 (10)	0.0159 (11)
S1	0.0352 (3)	0.0378 (3)	0.0362 (3)	−0.0018 (2)	−0.0033 (2)	−0.0015 (2)

### Geometric parameters (Å, °)

**Table:**

C1—C6	1.381 (3)	C9—H9	0.9300
C1—C2	1.389 (3)	C10—C11	1.386 (3)
C1—S1	1.772 (2)	C10—C14	1.489 (4)
C2—C3	1.369 (3)	C11—C12	1.384 (4)
C2—N2	1.478 (3)	C11—H11	0.9300
C3—C4	1.369 (4)	C12—C13	1.374 (3)
C3—H3	0.9300	C12—H12	0.9300
C4—C5	1.376 (4)	C13—H13	0.9300
C4—H4	0.9300	C14—H14A	0.9600
C5—C6	1.376 (3)	C14—H14B	0.9600
C5—H5	0.9300	C14—H14C	0.9600
C6—H6	0.9300	N1—S1	1.6374 (19)
C7—O3	1.205 (3)	N1—H1N	0.847 (16)
C7—N1	1.402 (3)	N2—O4	1.208 (3)
C7—C8	1.482 (3)	N2—O5	1.220 (3)
C8—C9	1.383 (3)	O1—S1	1.4159 (17)
C8—C13	1.389 (3)	O2—S1	1.4288 (16)
C9—C10	1.384 (3)		
			
C6—C1—C2	118.2 (2)	C11—C10—C14	122.2 (2)
C6—C1—S1	118.96 (17)	C12—C11—C10	120.8 (2)
C2—C1—S1	122.75 (16)	C12—C11—H11	119.6
C3—C2—C1	121.6 (2)	C10—C11—H11	119.6
C3—C2—N2	116.8 (2)	C13—C12—C11	120.7 (2)
C1—C2—N2	121.6 (2)	C13—C12—H12	119.6
C4—C3—C2	119.3 (2)	C11—C12—H12	119.6
C4—C3—H3	120.3	C12—C13—C8	119.3 (2)
C2—C3—H3	120.3	C12—C13—H13	120.3
C3—C4—C5	120.3 (2)	C8—C13—H13	120.3
C3—C4—H4	119.9	C10—C14—H14A	109.5
C5—C4—H4	119.9	C10—C14—H14B	109.5
C4—C5—C6	120.3 (2)	H14A—C14—H14B	109.5
C4—C5—H5	119.9	C10—C14—H14C	109.5
C6—C5—H5	119.9	H14A—C14—H14C	109.5
C5—C6—C1	120.3 (2)	H14B—C14—H14C	109.5
C5—C6—H6	119.8	C7—N1—S1	123.95 (16)
C1—C6—H6	119.8	C7—N1—H1N	120.4 (17)
O3—C7—N1	120.8 (2)	S1—N1—H1N	113.5 (17)
O3—C7—C8	125.01 (19)	O4—N2—O5	125.7 (2)
N1—C7—C8	114.15 (19)	O4—N2—C2	117.4 (2)
C9—C8—C13	119.5 (2)	O5—N2—C2	116.9 (2)
C9—C8—C7	121.99 (18)	O1—S1—O2	119.82 (11)
C13—C8—C7	118.5 (2)	O1—S1—N1	109.29 (10)
C8—C9—C10	121.8 (2)	O2—S1—N1	104.51 (10)
C8—C9—H9	119.1	O1—S1—C1	107.53 (10)
C10—C9—H9	119.1	O2—S1—C1	108.46 (10)
C9—C10—C11	117.8 (2)	N1—S1—C1	106.52 (10)
C9—C10—C14	120.0 (2)		
			
C6—C1—C2—C3	−1.2 (3)	C14—C10—C11—C12	−178.3 (2)
S1—C1—C2—C3	174.72 (19)	C10—C11—C12—C13	−1.7 (4)
C6—C1—C2—N2	178.5 (2)	C11—C12—C13—C8	−0.3 (4)
S1—C1—C2—N2	−5.6 (3)	C9—C8—C13—C12	2.0 (3)
C1—C2—C3—C4	2.0 (4)	C7—C8—C13—C12	−176.7 (2)
N2—C2—C3—C4	−177.7 (2)	O3—C7—N1—S1	−0.6 (3)
C2—C3—C4—C5	−1.4 (4)	C8—C7—N1—S1	−179.30 (15)
C3—C4—C5—C6	0.0 (4)	C3—C2—N2—O4	117.3 (3)
C4—C5—C6—C1	0.7 (4)	C1—C2—N2—O4	−62.4 (3)
C2—C1—C6—C5	−0.1 (3)	C3—C2—N2—O5	−61.8 (3)
S1—C1—C6—C5	−176.2 (2)	C1—C2—N2—O5	118.5 (3)
O3—C7—C8—C9	157.0 (2)	C7—N1—S1—O1	−51.6 (2)
N1—C7—C8—C9	−24.3 (3)	C7—N1—S1—O2	179.02 (18)
O3—C7—C8—C13	−24.3 (3)	C7—N1—S1—C1	64.3 (2)
N1—C7—C8—C13	154.3 (2)	C6—C1—S1—O1	16.5 (2)
C13—C8—C9—C10	−1.7 (3)	C2—C1—S1—O1	−159.39 (18)
C7—C8—C9—C10	176.87 (19)	C6—C1—S1—O2	147.47 (18)
C8—C9—C10—C11	−0.2 (3)	C2—C1—S1—O2	−28.4 (2)
C8—C9—C10—C14	180.0 (2)	C6—C1—S1—N1	−100.53 (19)
C9—C10—C11—C12	1.9 (3)	C2—C1—S1—N1	83.54 (19)

### Hydrogen-bond geometry (Å, °)

**Table:**

*D*—H···*A*	*D*—H	H···*A*	*D*···*A*	*D*—H···*A*
N1—H1N···O2^i^	0.85 (2)	2.43 (2)	3.232 (3)	157 (2)

Symmetry codes: (i) −*x*, −*y*, −*z*+1.
